# Supplementary material for: Single cell analyses identify a highly regenerative and homogenous human CD34+ hematopoietic stem cell population
Source: Nat Commun. 2022 Apr 19;13:2048. doi: 10.1038/s41467-022-29675-w (PMC9018830; doi:10.1038/s41467-022-29675-w)
Supplement: Supplementary file 1 — Supplementary Information [file 41467_2022_29675_MOESM1_ESM.pdf]

Single Cell Analyses Identify a Highly Regenerative and Homogenous Human CD34+ Hematopoietic Stem Cell Population

Fernando Anjos-Afonso, Florian Buettner, Syed Mian, Hefin Rhys, Jimena Perez-Lloret, Manuel Garcia-Albornoz, Linda Ariza-McNaughton and Dominique Bonnet

Supplementary Information

Supplementary Figures

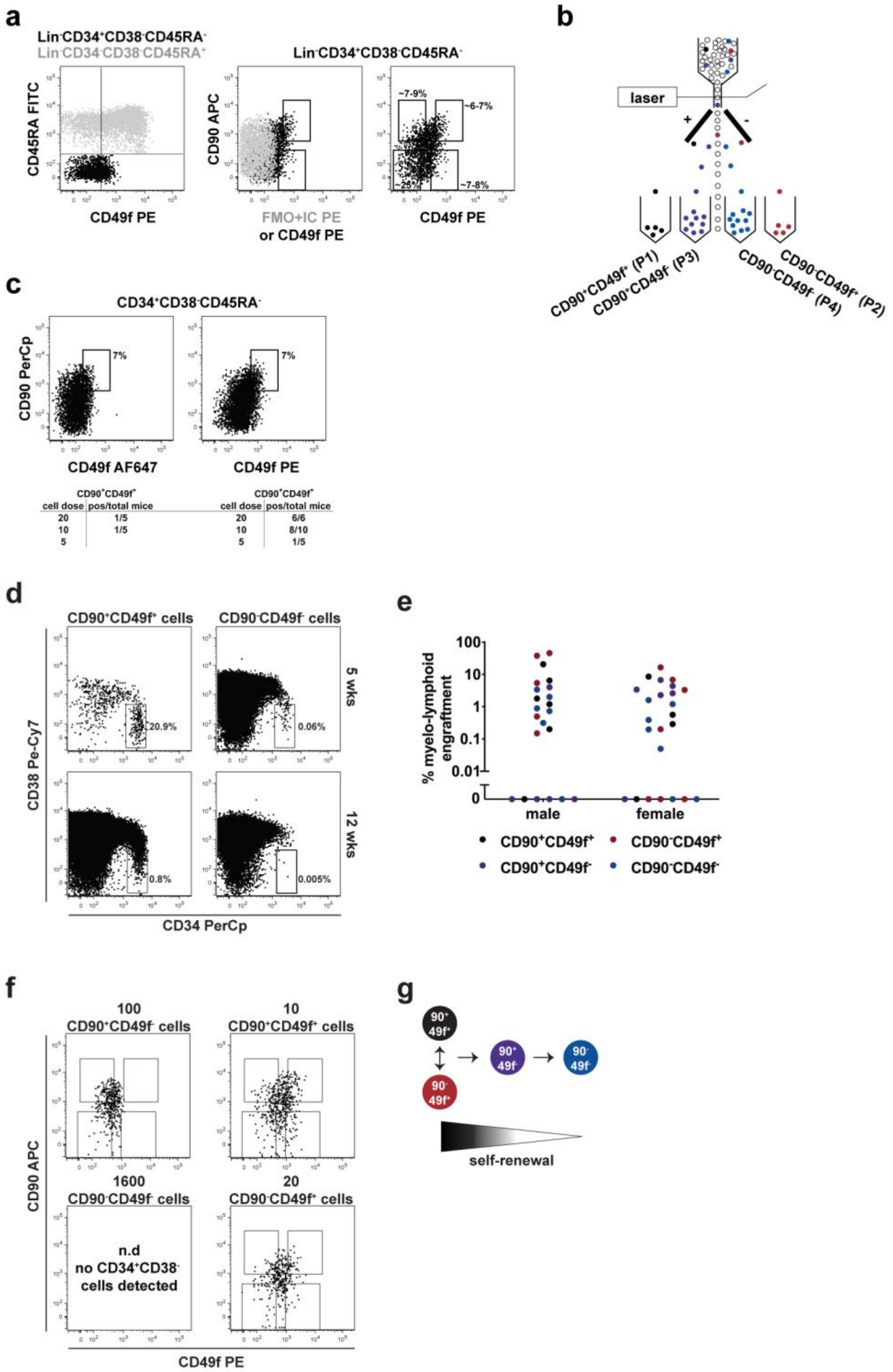

CD90+CD49f+ cell dose	pos/total mice
20	1/5
10	1/5
5	

CD90+CD49f+ cell dose	pos/total mice
20	6/6
10	8/10
5	1/5

Supplementary Fig. 1: Phenotypic and functional characterization of the four most primitive human HSPC populations.

a Representative FCM plots showing the expression of CD49f antigen on human cord blood lineage negative (Lin$^-$) cells. The left overlay plot shows that

the dim CD49f expression detected on $CD34^+CD38^-CD45RA^-$ HSPCs (black) was not due to the poor binding capacity of the anti-CD49f PE (clone GoH3) antibody used but owed to the weak CD49f expression on these cells, as the non-HSPCs (grey) were strongly stained. Fluorescent minus one (FMO) with an appropriate PE-conjugated isotype control antibody stain (grey in the middle overlay plot) was used to delineate the stringent gating of the four $CD90^{+or-}$ $CD49f^{+or-}$ populations (right plot). The values indicate the percentage range for each population within $CD34^+CD38^-CD45RA^-$ HSPCs used for cell sorting. **b** Schematic representation of the flow-sorting strategy. As an additional layer of quality control, during all first rounds of cell sorting the collection of the two $CD49f^+$ populations (P1 and P2) were assigned far apart from each other to minimize potential contamination between them. **c** PE and PE-Cy5 (not shown) but not AlexaFluor647 and FITC (not shown) conjugated anti-CD49f antibodies were able to reveal the dim CD49f expression on cord-blood $CD34^+$ HSPCs. As a result, $CD90^+CD49f^+$ cells sorted using anti-CD49f AlexaFluor647 antibodies mostly failed to engraft at limiting cell doses as compared to cells sorted using anti-CD49f PE antibodies. **d** Representative FCM plots depicting the frequency of human $CD34^+CD38^{lo/-}$ HSPCs found in the respective primary mouse BM at the indicated time points post-transplanted with ~1 $CD90^+CD49f^+$ or $CD90^-CD49f^-$ SRC. **e** Cumulative engraftment data derived from the four primitive populations assayed in NSG mice 12 wks after transplanting with ~1 SRC (data from **Fig. 1b**), showing no major difference in the level of engraftment between male and female recipients in the experimental settings used (n=10-13 mice/population). **f** Representative phenotype of the four sub-populations within $CD34^+CD38^{lo/-}CD45RA^-$HSPCs detected in the respective primary mouse BM engrafted at 12 wks with 1 SRC dose of the indicated cell populations. Cumulative data from all the mice at the indicated time points is represented in **Fig. 1d**. n.d: not detected. **g** Schematic representation of the hierarchical organization within the most primitive $CD34^+$ compartment supported by the data presented in **Fig. 1**. Source data are provided as a Source Data file.

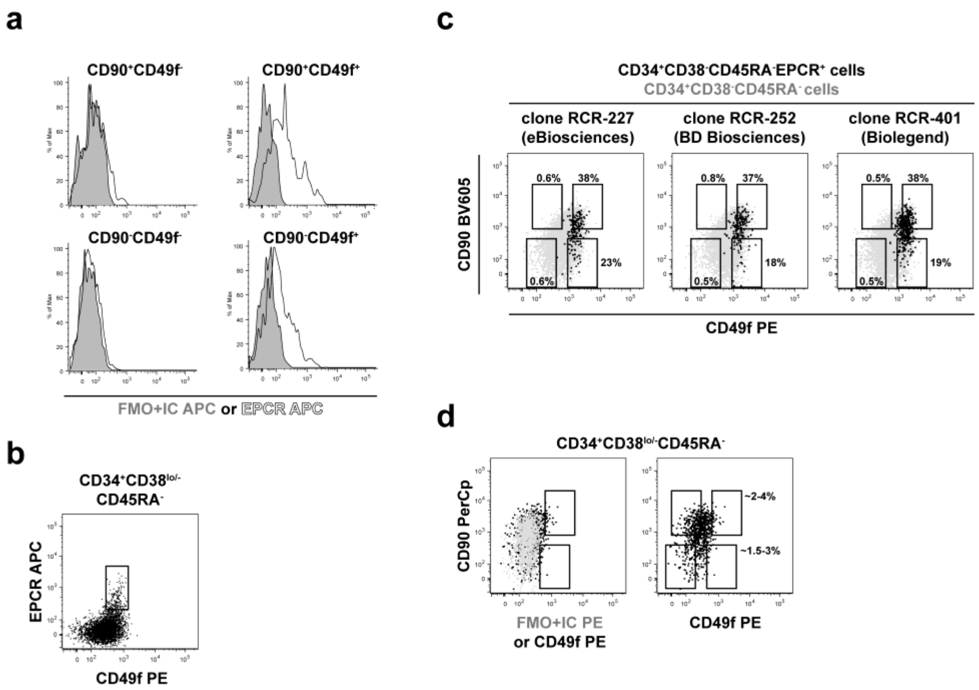

Supplementary Fig. 2: Phenotypic characterization of $EPCR^+$ cells.
a Representative overlay FCM histograms illustrating the expression of EPCR (open) on the four most primitive human $CD34^+$ HSPCs. In this example, $CD34^+$ cells were stained with the anti-CD201 APC clone RCR-227 or with an appropriate APC-conjugated isotype antibody (grey; as FMO stain) as negative control. **b** Representative FCM plot illustrating that almost all $EPCR^+$ cells in the $CD34^+C38^-CD45RA^-$ HSPC fraction express CD49f. **c** Representative overlay FCM plots showing that all the three clones highlighted $EPCR^+$ cells were mostly $CD90^+CD49f^+$ and $CD90^-CD49f^+$ cells. **d** An example of CD49f expression on adult BM $CD34^+$ cells by FCM analysis demonstrating almost negligible expression if this marker (black), as the fluorescent signal mostly overlapped with the signal derived from FMO with an appropriate PE-conjugated isotype control antibody stain (grey). Representative FCM plot illustrating the gating used to delineate the four populations based on CD90 and CD49f expressions on adult BM $CD34^+$ cells. The values indicate the percentage range for each population within $CD34^+CD38^-CD45RA^-$ HSPCs from 7 BM samples.

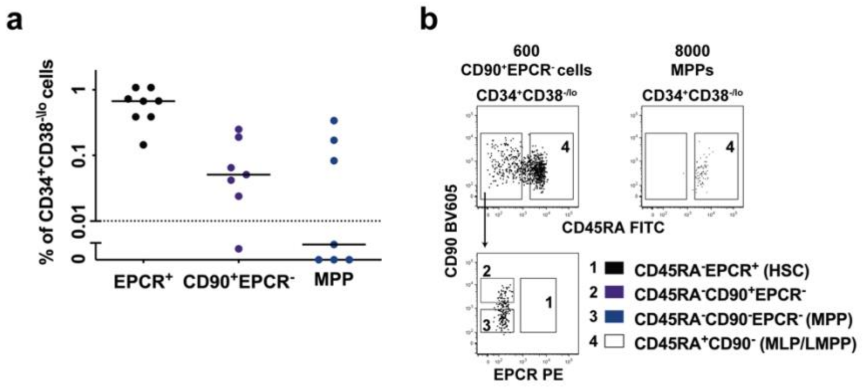

Supplementary Fig. 3: Contribution of CD90$^+$EPCR$^-$ cells to the different HSPCs *in vivo*.

a Frequency of CD34$^+$CD38$^{lo/-}$ HSPCs within human graft found in the BM of primary recipients transplanted with 15 EPCR$^+$ or 600 CD90$^+$EPCR$^-$ cells or 8000 MPPs (~5 SRCs). Mice with sufficient human HSPC graft (above the dotted line) were used for analysis in **Fig. 4c** (n=7-8 mice); median lines are shown. **b** Representative FCM plots illustrating the phenotype of the denoted sub-populations within CD34$^+$CD38$^{lo/-}$ HSPCs detected at 12 wks in the primary mouse bone marrow transplanted with CD90$^+$EPCR$^-$ progenitors or MPPs. The CD201 clone RCR-401 antibody was used in these experiments. Source data are provided as a Source Data file.

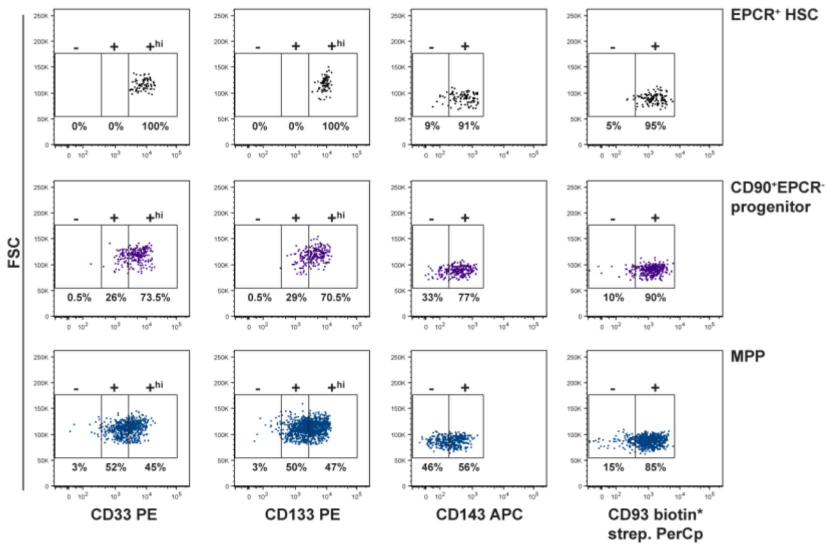

Supplementary Fig. 4: Expression of different stem cell associated antigens on the most primitive human CD34$^+$ HSPCs.

Representative FCM plots showing the expression of CD33, CD133 (AC133), CD143 (ACE2) and CD93 (C1qRp) on the denoted cell populations. The anti-CD201 clone RCR-227 and the anti-CD201 clone RCR-401 were used.

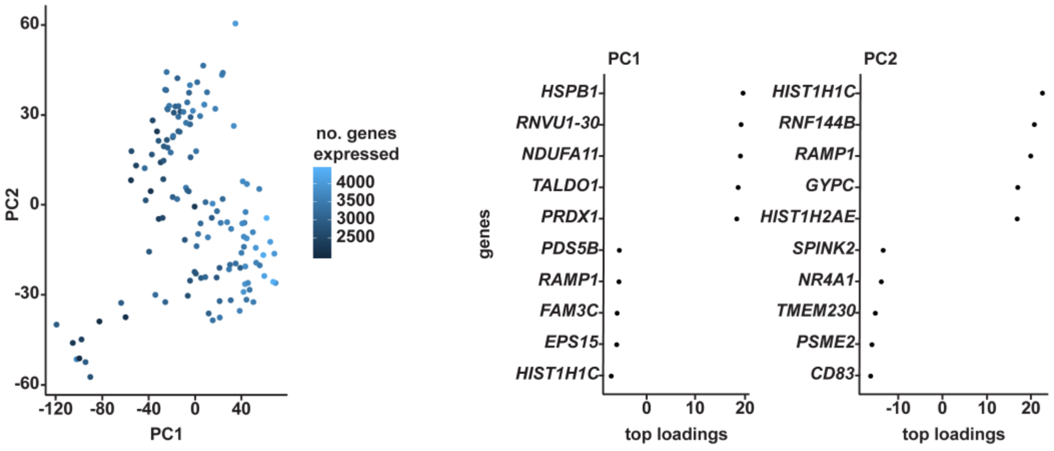

Supplementary Fig. 5: Single cell transcriptomic analyses of EPCR$^+$ HSCs.

Two-dimensional representation of the scRNA-seq performed in 135 single EPCR$^+$ HSCs (left graph) by PCA analysis. PCA visualization of the most variable genes in the EPCR$^+$ HSCs (right graphs). Source data are provided as a Source Data file.

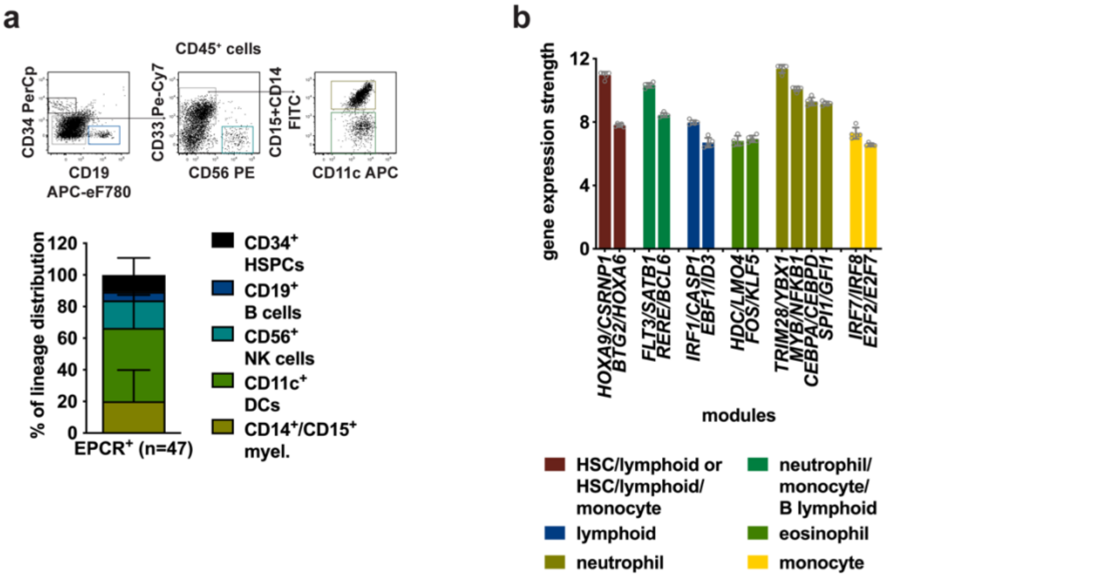

Supplementary Fig. 6: *In vitro* multipotency of EPCR$^+$ HSCs is supported by their transcriptomic wiring.

a Representative FCM plots (top) illustrating the different cell lineage outputs derived from 3 EPCR$^+$ HSCs in MS5-based culture (n=47). The focus was on myeloid vs lymphoid lineages: B lymphoid cells, CD19$^+$(CD34$^-$CD33$^-$); NK-like cells CD56$^+$(CD34$^-$CD33$^-$CD19$^-$); myeloid cells, CD14$^+$/CD15$^+$ (CD34$^-$CD33$^+$); myeloid-derived DC-like cells, CD11c$^+$CD33$^+$(CD34$^-$CD19$^-$). Cumulative data from all the individual cultures (n=47) is shown (bottom graph; from 3 independent experiments). **b** Expression strength of the genes associated with the denoted lineage-priming modules[8] detected in EPCR$^+$ HSCs (n=5). Bars are the mean values and error bars are the S.D for the no. of experiments performed. data are provided as a Source Data file.

Supplementary Table I

Detailed information of the reagents used in this study.

REAGENTS/RESOURCES	SOURCE	IDENTIFIER
**Antibodies (Flow Cytometry)**		
CD11c APC	BD Biosciences	B-ly6
CD14 FITC	BD Biosciences	M5E2
CD15 FITC	BD Biosciences	HI98
CD19 APC-eFluor780	Thermo Fisher Scientific/eBioscience	HIB19
CD19 FITC	BD Biosciences	HIB19
CD33 PE	BD Biosciences	WM53
CD33 PE-Cy7	Thermo Fisher Scientific/eBioscience	WM53
CD34 FITC	BD Biosciences	581
CD34 PerCp	BD Biosciences	8G12
CD38 PE-Cy7	Thermo Fisher Scientific/eBioscience	HB7
CD38 APC-eFluor780	Thermo Fisher Scientific/eBioscience	HB7
CD45 FITC	BD Biosciences	HI30
CD45 PerCp	BD Biosciences	HI30
CD45 APC	BD Biosciences	HI30
CD45RA FITC	BD Biosciences	HI100
CD45RA PE-Cy7	Thermo Fisher Scientific/eBioscience	HI100
CD49f PE	BD Biosciences	GoH3
CD49f AlexaFluor 647	BD Biosciences	GoH3
CD56 PE	BD Biosciences	B159
CD90 PerCp-eFluor710	Thermo Fisher Scientific/eBioscience	5E10
CD90 APC	Thermo Fisher Scientific/eBioscience	5E10
CD90 BV605	BD Biosciences	5E10
CD93/C1qRp Biotin	Biolegend	VIMD2
CD133 PE	Miltenyi Biotec	AC133
CD143 APC	BD Biosciences	BB9
CD201/EPCR PE	BD Biosciences	RCR-252
CD201/EPCR APC	BD Biosciences	RCR-252
CD201/EPCR PE	Biolegend	RCR-401
CD201/EPCR APC	Biolegend	RCR-401
CD201/EPCR APC	Thermo Fisher Scientific/eBioscience	RCR-227
**Chemicals and Recombinant Proteins for Cell Culture and *in vivo* Experiments**		
Ficoll-Paque	GE Healthcare Life Sciences	17-1440-02
EasySep CD34$^+$ Selection Kit II	StemCell Technologies	17856

EasySep Mouse/Human Chimera Enrichment kit	StemCell Technologies	19849
StemSep Human Progenitor Enrichment Kit	StemCell Technologies	14056
Collagen Solution	StemCell Technologies	04902
alpha-Minimum Essential media with nucleosides (αMEM)	Thermo Fisher Scientific/Gibco	22571038
MyeloCult™ H5100	StemCell Technologies	05150
StemSpan™ SFEM II	StemCell Technologies	09605
IL2	Peprotech	200-02
IL7	Peprotech	200-07
IL15	Peprotech	200-15
FLT3L	Peprotech	300-19
G-CSF	Peprotech	300-23
SCF	Peprotech	300-07
TPO	Peprotech	300-18
Immune Globulin Intravenous (IVIG; human)	Bio Products Laboratory (BPL)	Gammaplex 5%
Betamox 150 mg/ml suspension for injection	Norbrook	Betamox LA
4′,6-Diamidino-2-phenylindole dihydrochloride (DAPI)	Merck/Sigma-Aldrich	D8417
CellTrace™ Violet staining kit	Thermo Fisher Scientific	C34557
TMRE (tetramethylrhodamine ethyl ester)	BD Biosciences	564696
CellTitre-Glow 2.0 kit	Promega	G9241
Click-&-Go Plus 488 OPP Protein Synthesis Assay Kit	Clickchemistrytools	1493
Saponin	Merck-Sigma	47036
**Reagents and Materials for Molecular Biology**		
96-well skirted FrameStar PCR plates	4titude	4ti-0960
TrixonX-100 solution	Merck/Sigma-Aldrich	93443
RNAse inhibitor	Thermo Fisher Scientific/Ambion	AM2682
KAPA Stranded with RiboErase RNA-seq kit	KAPA Biosystems	KK8483
Kapa Dual-Indexed Adapters	KAPA Biosystems	KK8720
RNeasy Plus Micro Kit	Qiagen	74034
Agencourt AMPure XP beads	Beckman Coulter	A63880
Nextera™ XT DNA Sample Prep Kit	Illumina	FC-131-1096
C1™ Single-Cell Reagent Kit for mRNA Seq	Fluidigm	100-6201
Ethidium homodimer-1	Thermo Fisher Scientific/Invitrogen	E1169
Calcein AM	Thermo Fisher Scientific/Invitrogen	C3099

SMART-Seq v4 Ultra Low Input RNA Kit for C1 System	Takara	635026
**Software and Algorithms**		
FACSDiva v. 8.0.1.1	BD Biosciences	N/A
FlowJo v 9.96 and 10.6.2	FlowJo, LLC	N/A
Extreme Limiting Dilution Analysis (ELDA)	Hu and Smyth (2009)	http://bioinf.wehi.edu.au/software/elda/index.html
Prism 8.4.2	GraphPad Software	N/A
Adobe Illustrator 26.0.3	Adobe Inc.	N/A
DeSeq2 V1.28.1	Love *et al*., 2014	https://bioconductor.org/packages/release/bioc/html/DESeq2.html
GSEA 4.1.0	Subramanian *et al*., 2005	www.gsea-msigdb.org/gsea/index.jsp
STAR_2.4	Dobin *et al*., 2013	https://github.com/alexdobin/STAR
RSEM_1.2.8	Li *et al*., 2011	https://github.com/deweylab/RSEM
R 3.5.0	N/A	https://www.r-project.org
