# Supplementary material for: Single cell analyses identify a highly regenerative and homogenous human CD34+ hematopoietic stem cell population
Source: Nat Commun. 2022 Apr 19;13:2048. doi: 10.1038/s41467-022-29675-w (PMC9018830; doi:10.1038/s41467-022-29675-w)
Supplement: Supplementary file 3 — Description of Additional Supplementary Files [file 41467_2022_29675_MOESM3_ESM.pdf]

Description of Additional Supplementary Files

File name: Supplementary Data 1
Description: *In vivo* LDA data.
Tables with the *in vivo* limiting dilution assay data.

File name: Supplementary Data 2
Description: Bulk RNA-Seq., of the four human HSPC populations.
Tables with DE genes from the bulk RNA-seq analyses between the four $CD90^{+or-}CD49f^{+or-}$ HSPCs.

File name: Supplementary Data 3
Description: scRNA-Seq., $CD90^{+or-}CD49f^{+}$
Data analysis of the single-cell RNA-seq experiments between the two $CD49f^{+}$ HSC-enriched populations.

File name: Supplementary Data 4
Description: bulk RNA-Seq., $EPCR^{+}$vs$CD90^{+}EPCR^{-}$vsMPP
Tables with DE genes from the bulk RNA-seq analyses between the three most primitive human HSPC populations.
