# Supplementary material for: Single cell analyses identify a highly regenerative and homogenous human CD34+ hematopoietic stem cell population
Source: Nat Commun. 2022 Apr 19;13:2048. doi: 10.1038/s41467-022-29675-w (PMC9018830; doi:10.1038/s41467-022-29675-w)
Supplement: Supplementary file 8 — Reporting Summary [file 41467_2022_29675_MOESM8_ESM.pdf]

nature research

Corresponding author(s): Dominique Bonnet and Fernando Anjos-Afonso

Last updated by author(s): Feb 4, 2022

Reporting Summary

Nature Research wishes to improve the reproducibility of the work that we publish. This form provides structure for consistency and transparency in reporting. For further information on Nature Research policies, see our Editorial Policies and the Editorial Policy Checklist.

Statistics

For all statistical analyses, confirm that the following items are present in the figure legend, table legend, main text, or Methods section.

n/a	Confirmed	
☐	☒	The exact sample size (*n*) for each experimental group/condition, given as a discrete number and unit of measurement
☐	☒	A statement on whether measurements were taken from distinct samples or whether the same sample was measured repeatedly
☐	☒	The statistical test(s) used AND whether they are one- or two-sided<br>*Only common tests should be described solely by name; describe more complex techniques in the Methods section.*
☐	☒	A description of all covariates tested
☐	☒	A description of any assumptions or corrections, such as tests of normality and adjustment for multiple comparisons
☐	☒	A full description of the statistical parameters including central tendency (e.g. means) or other basic estimates (e.g. regression coefficient) AND variation (e.g. standard deviation) or associated estimates of uncertainty (e.g. confidence intervals)
☐	☒	For null hypothesis testing, the test statistic (e.g. F, t, r) with confidence intervals, effect sizes, degrees of freedom and P value noted<br>*Give P values as exact values whenever suitable.*
☒	☐	For Bayesian analysis, information on the choice of priors and Markov chain Monte Carlo settings
☒	☐	For hierarchical and complex designs, identification of the appropriate level for tests and full reporting of outcomes
☒	☐	Estimates of effect sizes (e.g. Cohen's *d*, Pearson's *r*), indicating how they were calculated

Our web collection on statistics for biologists contains articles on many of the points above.

Software and code

Policy information about availability of computer code

Data collection	FACS Diva v8 or above was used for cell sorting and collection.
Data analysis	FlowJo v9 or above; Prism v8.4 or above; ELDA (http://bioinf.wehi.edu.au/software/elda/index.html); Adobe Illustrator 28.0.3; bioinformatics packages: DeSeq2 V1.28.1, STAR_2.4, RSEM_1.2.8; R 3.5.0; GSEA 4.1.0 (details in Supplementary Information).

For manuscripts utilizing custom algorithms or software that are central to the research but not yet described in published literature, software must be made available to editors and reviewers. We strongly encourage code deposition in a community repository (e.g. GitHub). See the Nature Research guidelines for submitting code & software for further information.

Data

Policy information about availability of data

All manuscripts must include a data availability statement. This statement should provide the following information, where applicable:

- Accession codes, unique identifiers, or web links for publicly available datasets
- A list of figures that have associated raw data
- A description of any restrictions on data availability

All RNA-seq data that support the findings have been deposited to the Gene Expression Omnibus with the following accession numbers: GSE155174, GSE154263, GSE154588, GSE154931 and GSE197079. Source data are available for Figs. 1 to 7 and Supplementary Figs. 1, 3, 5 and 6. Wikipathways databased was used.

Field-specific reporting

Please select the one below that is the best fit for your research. If you are not sure, read the appropriate sections before making your selection.

☒ Life sciences ☐ Behavioural & social sciences ☐ Ecological, evolutionary & environmental sciences

For a reference copy of the document with all sections, see nature.com/documents/nr-reporting-summary-flat.pdf

Life sciences study design

All studies must disclose on these points even when the disclosure is negative.

Sample size	For animal work: a) non LDA experiments, sample size (>8 animals), b) LDA experiments, sample size (4 or more animals/cell dose; 4 or more cell doses/group) were chosen to gain sufficient confidence between groups. For RNA-seq., population level (n=3) and single cells (>100 to 500) were the maximum feasible sizes for each set of experiments were performed to give confidence between groups; for other in vitro experiments: 3 to 4 independent experiments were performed to reach enough power and confidence to reach statistical significance when using two-tailed paired or unpaired t-tests (but no prior sample size calculation was used).
Data exclusions	Single cells which failed standard QC for RNA-seq were excluded from analysis.
Replication	All replication attempts were successful: in vitro assays (3 to 4); population level RNA-seq (3 to 5)
Randomization	Both male and female mice were used in equal numbers in each experiment to avoid bias. But no explicit randomization was performed as the genetic background of the mice was equivalent in all animals. For other experiments randomization was not necessary and relevant to the experiments.
Blinding	No explicit blinding was performed. All chimerism calls were based on hard data, and thus blinding isn't able to change the result.

Reporting for specific materials, systems and methods

We require information from authors about some types of materials, experimental systems and methods used in many studies. Here, indicate whether each material, system or method listed is relevant to your study. If you are not sure if a list item applies to your research, read the appropriate section before selecting a response.

Materials & experimental systems

n/a	Involved in the study	
☐	☒	Antibodies
☐	☒	Eukaryotic cell lines
☒	☐	Palaeontology and archaeology
☐	☒	Animals and other organisms
☐	☒	Human research participants
☒	☐	Clinical data
☒	☐	Dual use research of concern

Methods

n/a	Involved in the study	
☒	☐	ChIP-seq
☐	☒	Flow cytometry
☒	☐	MRI-based neuroimaging

Antibodies

Antibodies used	Antibodies were purchased from the indicated companies and based on company validation for use in flow cytometry (see Methods section for details).<br>BD Biosciences: CD11c APC (B-ly6); CD14 FITC (M5E2); CD15 FITC (HI98); CD19 FITC (HIB19); CD33 PE (WM53); CD34 FITC (581); CD34 PerCp (8G12); CD45 FITC (HI30); CD45 PerCp (HI30); CD45 APC (HI30); CD45RA FITC (HI100); CD49f PE (GoH3); CD49f AlexaFluor 647 (GoH3); CD56 PE (B159); CD90 BV605 (5E10); CD143 APC (BB9); CD201/EPCR PE (RCR-252); CD201/EPCR APC (RCR-252).<br><br>Thermo Fisher Scientific/eBioscience: CD19 APC-eFluor780 (HIB19); CD33 PE-Cy7 (WM53); CD38 PE-Cy7 (HB7); CD38 APC-eFluor780 (HB7); CD45RA PE-Cy7 (HI100); CD90 PerCp (5E10); CD90 APC (5E10); CD93 (VIMD2); CD201/EPCR APC (RCR-227).<br><br>Biolegend: CD201/EPCR PE (RCR-401); CD201/EPCR APC (RCR-401).<br><br>Miltenyi Biotec: CD133 PE (AC133).
Validation	All antibodies were validated according to respective manufacturer's information:<br>CD11c APC (https://www.bdbiosciences.com/en-us/products/reagents/flow-cytometry-reagents/research-reagents/single-color-antibodies-ruo/apc-mouse-anti-human-cd11c.559877)<br><br>CD14 FITC: https://www.bdbiosciences.com/en-us/products/reagents/flow-cytometry-reagents/research-reagents/single-color-

antibodies-ruo/fitc-mouse-anti-human-cd14.555397

CD15 FITC: https://www.bdbiosciences.com/en-us/products/reagents/flow-cytometry-reagents/research-reagents/single-color-antibodies-ruo/fitc-mouse-anti-human-cd15.555401

CD19 FITC: https://www.bdbiosciences.com/en-us/products/reagents/flow-cytometry-reagents/research-reagents/single-color-antibodies-ruo/fitc-mouse-anti-human-cd19.555412

CD33 PE: https://www.bdbiosciences.com/en-us/products/reagents/flow-cytometry-reagents/research-reagents/single-color-antibodies-ruo/pe-mouse-anti-human-cd33.555450

CD34 FITC: https://www.bdbiosciences.com/en-us/products/reagents/flow-cytometry-reagents/research-reagents/single-color-antibodies-ruo/fitc-mouse-anti-human-cd34.555821

CD34 PerCp: https://www.bdbiosciences.com/en-us/products/reagents/flow-cytometry-reagents/clinical-diagnostics/single-color-antibodies-asr-ivd-ce-ivd/cd34-percp.340666

CD45 FITC: https://www.bdbiosciences.com/en-us/products/reagents/flow-cytometry-reagents/research-reagents/single-color-antibodies-ruo/fitc-mouse-anti-human-cd45.555482

CD45 PerCp: https://www.bdbiosciences.com/en-us/products/reagents/flow-cytometry-reagents/clinical-diagnostics/single-color-antibodies-asr-ivd-ce-ivd/cd45-percp.345809

CD45 APC: https://www.bdbiosciences.com/en-us/products/reagents/flow-cytometry-reagents/research-reagents/single-color-antibodies-ruo/apc-mouse-anti-human-cd45.555485

CD45RA FITC: https://www.bdbiosciences.com/en-us/products/reagents/flow-cytometry-reagents/research-reagents/single-color-antibodies-ruo/fitc-mouse-anti-human-cd45ra.555488

CD49f PE: https://www.bdbiosciences.com/en-us/products/reagents/flow-cytometry-reagents/research-reagents/single-color-antibodies-ruo/pe-rat-anti-human-cd49f.555736

CD49f AlexaFluor647: https://www.bdbiosciences.com/en-us/products/reagents/flow-cytometry-reagents/research-reagents/single-color-antibodies-ruo/alexa-fluor-647-rat-anti-human-cd49f.562473

CD56 PE: https://www.bdbiosciences.com/en-us/products/reagents/flow-cytometry-reagents/research-reagents/single-color-antibodies-ruo/pe-mouse-anti-human-cd56-ncam-1.555516

CD90 BV605: https://www.bdbiosciences.com/en-us/products/reagents/flow-cytometry-reagents/research-reagents/single-color-antibodies-ruo/bv605-mouse-anti-human-cd90.747750

CD143 APC: https://www.bdbiosciences.com/en-us/products/reagents/flow-cytometry-reagents/research-reagents/single-color-antibodies-ruo/apc-mouse-anti-human-cd143.557929

CD201 PE: https://www.bdbiosciences.com/en-us/products/reagents/flow-cytometry-reagents/research-reagents/single-color-antibodies-ruo/pe-rat-anti-human-cd201.557950

CD201 APC: https://www.bdbiosciences.com/en-us/products/reagents/flow-cytometry-reagents/research-reagents/single-color-antibodies-ruo/apc-rat-anti-human-cd201.563622

CD19 Alexa-eFluor780: https://www.thermofisher.com/antibody/product/CD19-Antibody-clone-HIB19-Monoclonal/47-0199-42

CD33 PE-Cy7: https://www.thermofisher.com/antibody/product/CD33-Antibody-clone-WM-53-WM53-Monoclonal/25-0338-42

CD38 PE-Cy7: https://www.thermofisher.com/antibody/product/CD38-Antibody-clone-HIT2-Monoclonal/25-0389-42

CD38 Alexa-eFluor780: https://www.thermofisher.com/antibody/product/CD38-Antibody-clone-HIT2-Monoclonal/47-0389-42

CD45RA PE-Cy7: https://www.thermofisher.com/antibody/product/CD45RA-Antibody-clone-HI100-Monoclonal/25-0458-42

CD90 PerCp-eFluor 710: https://www.thermofisher.com/antibody/product/CD90-Thy-1-Antibody-clone-eBio5E10-5E10-Monoclonal/46-0909-42

CD90 APC: https://www.thermofisher.com/antibody/product/CD90-Thy-1-Antibody-clone-eBio5E10-5E10-Monoclonal/17-0909-42

CD201 APC: https://www.thermofisher.com/antibody/product/CD201-EPCR-Antibody-clone-RCR-227-Monoclonal/17-2018-42

CD93 Biotin: https://www.biolegend.com/en-gb/products/biotin-anti-human-cd93-antibody-5254

CD201 PE: https://www.biolegend.com/en-gb/products/pe-anti-human-cd201-epcr-antibody-7240

CD201 APC: https://www.biolegend.com/en-gb/products/apc-anti-human-cd201-epcr-antibody-7693

CD133 PE: https://www.miltenyibiotec.com/GB-en/products/cd133-1-antibody-anti-human-ac133.html#pe:100-tests-in-200-ul

Eukaryotic cell lines

Policy information about cell lines

Cell line source(s)	MS5 stroma cell line (DSMZ; cat. no: ACC441).
Authentication	MS5 cell line was not authenticated as it was purchased from DSMZ.
Mycoplasma contamination	MS5 stroma cell line was tested negative using different methods (broth/agar and DAPI staining).
Commonly misidentified lines (See ICLAC register)	Cell line not listed in the ICLAC website.

Animals and other organisms

Policy information about studies involving animals; ARRIVE guidelines recommended for reporting animal research

Laboratory animals	Males and Females (8-12 wks old) NOD.Cg-Prkdcscid Il2rgtm1Wjl/SzJ (NSG) mice were used as transplant recipients.
Wild animals	No wild animals were used.
Field-collected samples	No field-collected samples were used.
Ethics oversight	All animal experiments were performed under the U.K Home Office project licence (70/8904) in accordance with The Francis Crick Institute animal ethics committee guidance.

Note that full information on the approval of the study protocol must also be provided in the manuscript.

Human research participants

Policy information about studies involving human research participants

Population characteristics	All cords were collected from normal full-term deliveries, and thus are by default age-matched. Human BM were purchased from Stem Cell Technologies from males and female donors aged 28-50 years old.
Recruitment	Cord-bloods were pooled from different donors to avoid bias and human marrows were selected from late-young to early-middle-age adults to minimize potential variability due to aging.
Ethics oversight	Cord blood was obtained after informed consent from the Royal London Hospital (REC: 06/Q0604/110) and University of Hospitals of Wales (REC: 06/WSE03/6). The protocols were approved by their respective ethical committee.

Note that full information on the approval of the study protocol must also be provided in the manuscript.

Flow Cytometry

Plots

Confirm that:

- [x] The axis labels state the marker and fluorochrome used (e.g. CD4-FITC).
- [x] The axis scales are clearly visible. Include numbers along axes only for bottom left plot of group (a 'group' is an analysis of identical markers).
- [x] All plots are contour plots with outliers or pseudocolor plots.
- [x] A numerical value for number of cells or percentage (with statistics) is provided.

Methodology

Sample preparation	See Method section for details for the preparation of all samples.
Instrument	Sorts were performed with a BD FACSAria™ Fusion or III. For flow cytometry analyses a BD LSRFortessa™ was used.
Software	FACS Diva v8 or above was used for cell sorting and collection. FlowJo v9 or above was used for analyses.
Cell population abundance	To isolate the four CD90+or-CD49f+or- populations, cells were double-sorted with purities attaining >99%; To isolate other cell populations, when possible to check, purities were >98%.

Gating strategy

Gates were set based on a combination of the unstained sample (for negative gating) and Fluorescent-Minus-One (FMO) controls for channels where positivity could be ambiguous. Examples gating can be seen throughout the manuscript.

☒ Tick this box to confirm that a figure exemplifying the gating strategy is provided in the Supplementary Information.
